# Trans-Ethnic Mapping of *BANK1* Identifies Two Independent SLE-Risk Linkage Groups Enriched for Co-Transcriptional Splicing Marks

**DOI:** 10.3390/ijms19082331

**Published:** 2018-08-08

**Authors:** Manuel Martínez-Bueno, Nina Oparina, Mikhail G. Dozmorov, Miranda C. Marion, Mary E. Comeau, Gary Gilkeson, Diane Kamen, Michael Weisman, Jane Salmon, Joseph W. McCune, John B. Harley, Robert Kimberly, Judith A. James, Joan Merrill, Courtney Montgomery, Carl D. Langefeld, Marta E. Alarcón-Riquelme

**Affiliations:** 1 GENYO, Centre for Genomics and Oncological Research: Pfizer, University of Granada, Andalusian Government, PTS, 18016 Granada, Spain; manuel.martinez@genyo.es; 2 Unit of Chronic Inflammatory Diseases, Institute for Environmental Medicine, Karolinska Institutet, 171 67 Solna, Sweden; nina.oparina@ki.se; 3 Department of Biostatistics, Virginia Commonwealth University, Richmond, VA 23284, USA; mikhail.dozmorov@vcuhealth.org; 4 Center for Public Health Genomics, Wake Forest School of Medicine, Winston-Salem, NC 27157, USA; mimarion@wakehealth.edu (M.C.M.); mcomeau@wakehealth.edu (M.E.C.); clangefe@wakehealth.edu (C.D.L.); 5 Division of Rheumatology, Medical University of South Carolina, Charleston, SC 29425, USA; gilkeson@musc.edu (G.G.); kamend@musc.edu (D.K.); 6 Division of Rheumatology, Cedars-Sinai Medical Center, Los Angeles, CA 90048, USA; weisman@cshs.org; 7 Hospital for Special Surgery, New York, NY 10021, USA; jes2002@med.cornell.edu; 8 Department of Internal Medicine, University of Michigan, Ann Arbor, MI 48109, USA; jmccune@umich.edu; 9 Cincinnati Children’s Hospital Medical Center, OH and US Department of Veterans Affairs Medical Center, Cincinnati, OH 45229, USA; john.harley@cchmc.org; 10 School of Medicine, University of Alabama at Birmingham, Birmingham, AL 35205, USA; rpk@uab.edu; 11 Arthritis and Clinical Immunology and Clinical Pharmacology Programs, Oklahoma Medical Research Foundation, Oklahoma City, OK 73104, USA; judith-james@omrf.org (J.A.J.); Joan-Merrill@omrf.org (J.M.); courtney-montgomery@omrf.org (C.M.)

**Keywords:** autoimmune disorders, systemic lupus erythematosus, genetics, *BANK1*, transethnic genetic studies

## Abstract

*BANK1* is a susceptibility gene for several systemic autoimmune diseases in several populations. Using the genome-wide association study (GWAS) data from Europeans (EUR) and African Americans (AA), we performed an extensive fine mapping of ankyrin repeats 1 (*BANK1*). To increase the SNP density, we used imputation followed by univariate and conditional analysis, combined with a haplotypic and expression quantitative trait locus (eQTL) analysis. The data from Europeans showed that the associated region was restricted to a minimal and dependent set of SNPs covering introns two and three, and exon two. In AA, the signal found in the Europeans was split into two independent effects. All of the major risk associated SNPs were eQTLs, and the risks were associated with an increased *BANK1* gene expression. Functional annotation analysis revealed the enrichment of repressive B cell epigenomic marks (EZH2 and H3K27me3) and a strong enrichment of splice junctions. Furthermore, one eQTL located in intron two, rs13106926, was found within the binding site for RUNX3, a transcriptional activator. These results connect the local genome topography, chromatin structure, and the regulatory landscape of *BANK1* with co-transcriptional splicing of exon two. Our data defines a minimal set of risk associated eQTLs predicted to be involved in the expression of *BANK1* modulated through epigenetic regulation and splicing. These findings allow us to suggest that the increased expression of *BANK1* will have an impact on B-cell mediated disease pathways.

## 1. Introduction

The B cell adaptor with the ankyrin repeats 1 (*BANK1*) gene is a susceptibility gene for the systemic autoimmune diseases of systemic lupus erythematosus, rheumatoid arthritis, and systemic sclerosis. BANK1 is an adaptor molecule of 795aa in its full-length form (FL), and has two, less abundant smaller isoforms, one of which lacks the second exon (D2) [1]. The protein is expressed primarily in mature B cells and, to a lesser extent, in myeloid and plasmacytoid dendritic cells (pDCs). BANK1 protein has 23 tyrosines prone to phosphorylation, and it is extensively phosphorylated following the B cell receptor cross-linking [2]. BANK1 was first identified as a partner of the tyrosine kinase LYN2, and a knock-out mouse for BANK1 showed an increased CD40-dependent IgM production, germinal center formation, and increased activation of Akt [3]. It was therefore suggested that BANK1 could function as an inhibitor of B cell activation. It was recently shown that BANK1 shares, with other molecules, the presence of a conformational toll/IL-1 receptor domain (TIR), and this domain is coded by exon two; therefore, BANK1 could possibly play a role in toll-like receptor (TLR) signaling [4], important for autoimmunity. Supporting such a role for BANK1, we showed that a deficiency of BANK1 in the mouse leads to a reduction in the activity of the mitogen activation of proliferation kinase (MAPK) p38, which specifically reduces the activation of the translation initiation complex eIF4E, leading to a reduced production of IL-6, following stimulation with a TLR9 agonist (CpG) [5]. More recently, we demonstrated that crosses between a lupus model and the *BANK1*−/− mice improve several disease B cell phenotypes; primarily, there is a reduction in the production of the total IgG and IgG anti-dsDNA antibodies, a reduction in serum IL-6 and BAFF, and reduced pSTAT1 signaling at Tyrosine 701, following the TLR7-agonist stimulation. These data suggest a role for BANK1 in the production of IgG dependent on STAT1. On the other hand, BANK1 has no direct role in IFN JAK-STAT signaling, but instead, its signaling function is through TLR, reducing the expression of Ifnb and Ifna2, as well as reducing the subsequent autocrine uptake by the IFN alpha receptor (IFNAR) in the *BANK1*−/− mice [6]. Thus, the increased expression of the FL *BANK1* isoform should increase the risk for systemic lupus erythematosus (SLE). How the expression of *BANK1* is regulated is presently not known.

The genetic association with an autoimmune disease involved two polymorphisms, a branch point site SNP (rs17266594), suggested to be involved in splicing efficiency and associated with increased expression of the full-length isoform of *BANK1*, and a coding R61H variant identified through SNP rs10516487 located in exon two. Both of the SNPs were in strong linkage disequilibrium. Only the highly expressed full-length isoform contained the risk allele for *BANK1* (R61) [1]. We later showed R61H to have a potential effect in splicing and protein aggregation [7]. Using a minigene and a reporter assay, we showed that the protective variant, encoding 61H, was not only influencing the protein function, but that it also disturbed the splicing of exon two by creating a binding of the splicing protein SRp40, therefore increasing the quantity of the D2 isoform of *BANK1* and reducing that of the FL [7].

In order to determine, with precision, the location of the genetic association of *BANK1*, and to investigate whether the SNPs within *BANK1* could affect its expression, we performed a high-density analysis of *BANK1*, through genotyping and imputation, combined with eQTL analyses using all of the databases available, in a set of over 10,200 individuals of European and African American ancestries, combined with ENCODE (Encyclopedia of DNA Elements) track analysis.

## 2. Results

### BANK1 Transethnic Finemapping

**Figure 1:**
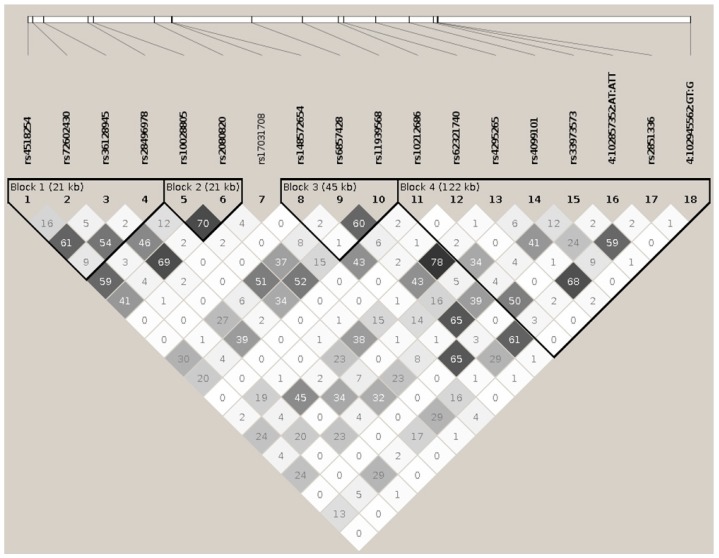
Haplotypic architecture of the European (EUR) ankyrin repeats 1 (*BANK1*) gene. Plot representing pairwise *r*^2^ values. Eighteen tagging markers captured all 307 (100%) alleles at *r*^2^ ≥ 0.8, representing their 18 respective linkage disequilibrium groups. These 18 markers are organized in four haplotypic blocks. The calculation of haplotypic blocks was made using D’.

**Figure 2:**
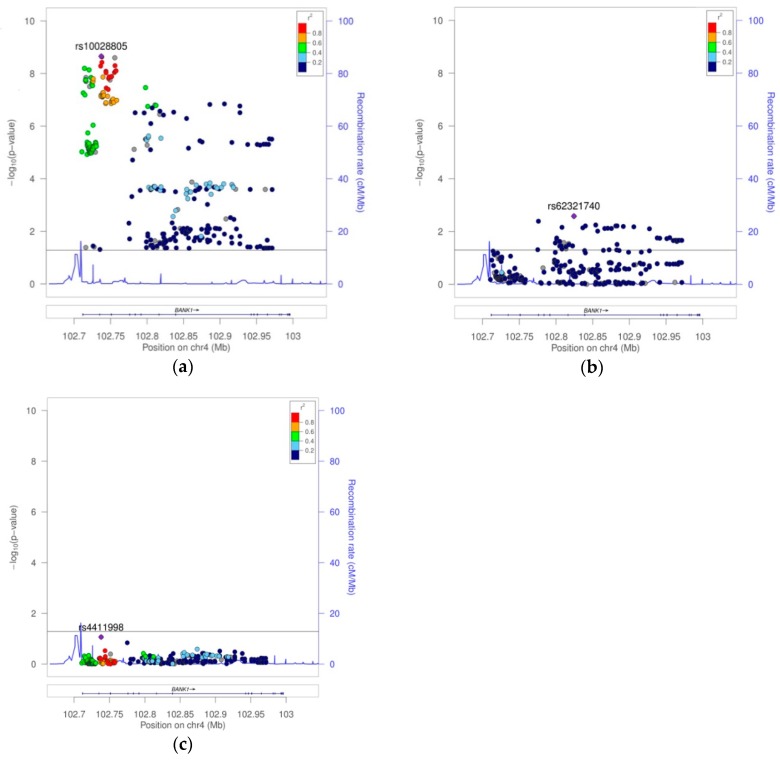
Plot of 307 markers with association *p* value < 0.05 in the EUR sample. (**a**) Case–control association analysis result. (**b**) Case–control association analysis conditioning on the best-hit rs10088205. (**c**) Case–control association analysis result conditioning on markers rs10088205, rs148572654, rs62321740, and 4:102945562:GT:G.

**Figure 3:**
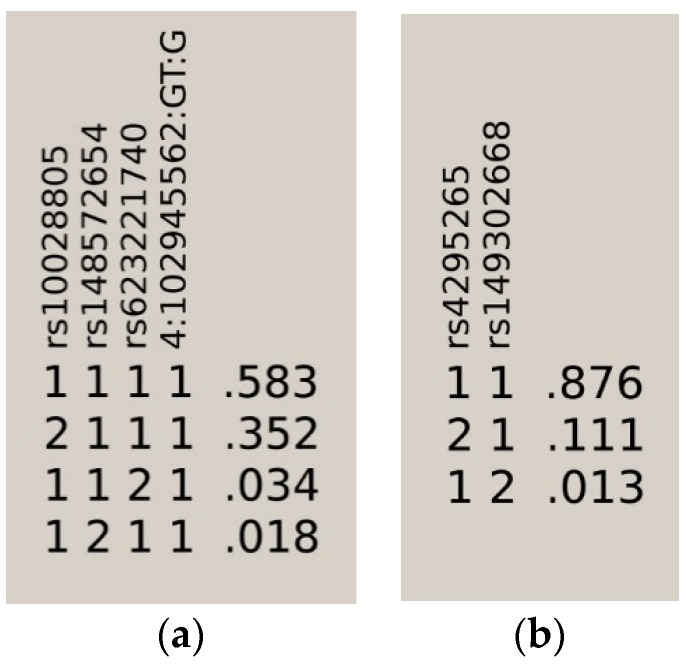
Haplotypes associated with the affected phenotype in the (**a**) EUR sample and (**b**) AA sample.

Using the high quality filtered genome-wide data from the European and African American genome-wide association scans, we performed a univariate genetic logistic regression association analysis of *BANK1* using an additive model. In Europeans, 1133 imputed markers mapped on *BANK1* were tested for association. We selected a set of 307 markers with a *p* value lower than 0.05 (Supplemental Table S1A and Supplemental Figure S1A). Eighteen tagging markers captured all 307 (100%) alleles at *r*^2^ ≥0.8, representing the 18 respective linkage disequilibrium groups organized in four haplotypic blocks. Only one marker remained as an independent signal, rs17031708 (Figure 1, Table 1 and Supplemental Table S1A). The best hit for association was the SNP rs10028805, (haplotypic block two, and linkage disequilibrium group five), with OR = 0.8177, confidence interval (CI) = 95% (0.7656, 0.8735), and a Pcorr = 2.22E−09 (Figure 2a, Table 1, and Supplemental Table S1A). Conditioning on rs10028805, the association disappeared, except that of the five low frequency variants (MAF < 5%), rs148572654, rs10212686, rs62321740, 4:102857352:AT:ATT, and 4:102945562:GT:G (Table 1 and Figure 2b), representing linkage groups 8, 11, 12, 16, and 19, respectively. Only by conditioning on rs10028805 and three of these low frequency variants, namely, rs148572654, rs62321740, and 4:102945562:GT:G, did all of the signals of association in *BANK1* disappear (Table 1, Supplemental Table S1A, and Figure 2c). In addition, these four markers constituted the haplotypes significantly associated with the affected phenotype. The best was the allelic combination ‘1111’, corresponding to a risk haplotype with frequencies of 62% in the cases and 56% in the controls, and a value of *p* = 7.64E−13 (Table 2, Figure 3a).

**Figure 4:**
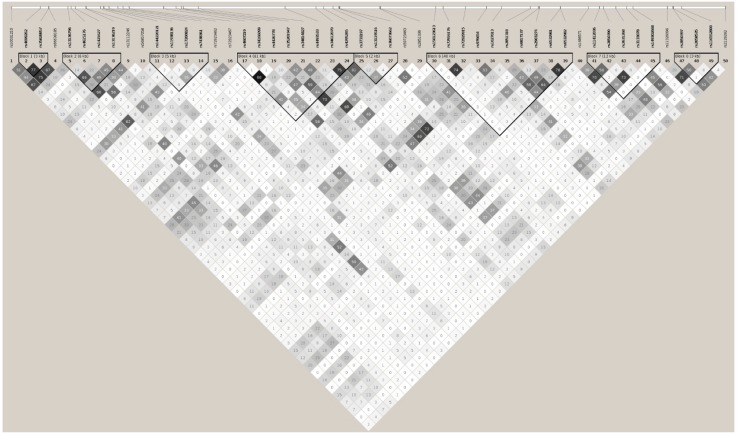
Haplotypic architecture of *BANK1* in AA. Plot representing pairwise r² values. Fifty tagging markers captured all 260 (100%) alleles at *r*^2^ ≥ 0.8, representing their 50 respective linkage groups in the AA sample. These 50 markers were organized in 8 haplotypic blocks and 11 independent signals. Calculation of haplotypic blocks was made using D’.

**Figure 5:**
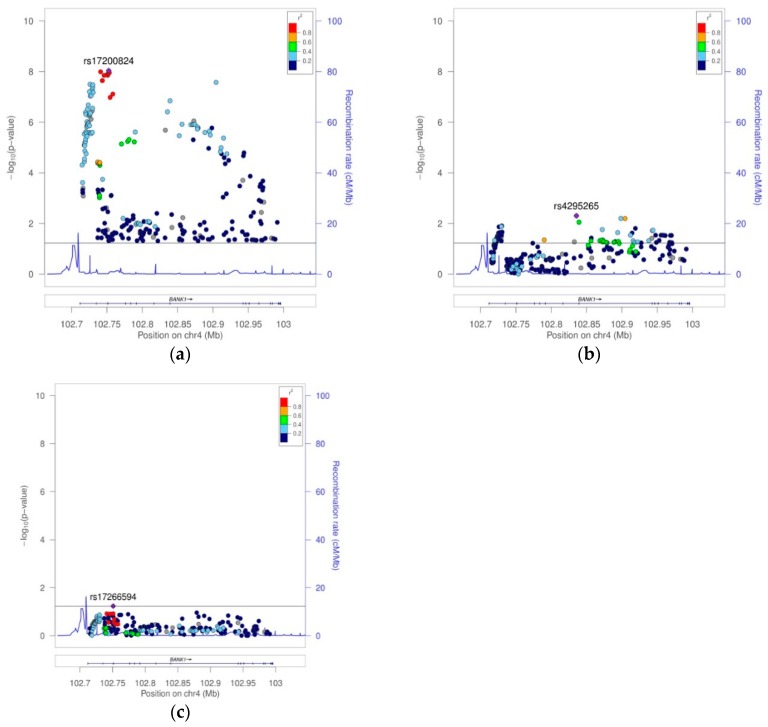
Plot of 260 markers with association *p* values < 0.05 in AA. (**a**) Case–control association analysis result. (**b**) Case–control association analysis result conditioning on best-hit rs17200824. (**c**) Case–control association analysis result conditioning on markers rs17200824, rs4295265, and rs149302668.

In African Americans, 1752 imputed SNPs were mapped on *BANK1*, of which 260 showed association test *p* values lower than 0.05 (Supplemental Table S1B and Supplemental Figure S1B). Running the tagger tool, a set of 50 markers captured all 260 (100%) alleles at *r*^2^ ≥0.8, representing their 50 respective linkage disequilibrium groups (Table 3 and Supplemental Table S1B). These 50 tagging markers were organized in 8 haplotypic blocks and 11 independent signals (Figure 4). The best hit was the SNP rs17200824 with OR = 0.6787, CI = 95% (0.5947, 0.7747), and a Pcorr = 9.24E−09. This best-hit was in linkage group 13, the same as the branch-point site SNP rs17266594 and rs10516487 (R61H) (Figure 5a, Table 3, and Supplemental Table S1B). After conditioning on rs17200824, the association signals remained in 11 of the tagging markers (Figure 5b, Table 3, and Supplemental Table S1B). Only after the conditioning on rs17200824 on two of these 11 variants, rs4295265 and rs149302668, had all of the signals on the *BANK1* association disappeared (Table 3, Supplemental Table S1B and Figure 5c). In addition, these two markers constituted haplotypes that are significantly associated with the affected phenotype; the best was the allelic combination ‘11’, corresponding to a risk haplotype with frequencies of 90% in the cases and 84.6% in the controls, and a value of Pcorr = 3.17E−08 (Table 4 and Figure 3b). It is noted that rs4295265 (Pcorr = 3.89E–07) was located on intron seven, at 2,319 bp upstream to the exonic SNP rs3733197 (A 353 T, in the ankyrin domain) (Pcorr = 1.44E−07), described as being associated to SLE1 and sharing the same haplotypic block eight (pairwise *r*^2^ = 0.75). The SNP rs149302668, located on intron 11, was a low frequency variant (MAF = 0.01348).

In EUR, markers significantly associated with SLE were located to haplotypic blocks one and two, including intron one and intron two–exon two–intron three. In the first haplotypic block, covering intron one, the markers were organized in linkage disequilibrium groups one, two, and three. Group one showed the best signal of association in the block with rs4518254 (Pcorr = 6.36E–09) as a best hit. The second haplotypic block included two linkage disequilibrium groups, five and six, covering intron two–exon two–intron three. Group five contained rs10028805 (intron2) (Pcorr = 2.22E–09) and rs10516486 (L98L, exon2). For group six, rs13106926 (intron2) (Pcorr = 1.36E–08) was the best hit; this group also contained the branch-point site, SNP rs17266594 (intron1), and rs10516487 (R61H, exon two) [1].

After conditioning on the best-hit, rs10028805, all of the signals of association for the common variation (MAF > 5%) in *BANK1* disappeared; hence, rs10028805 would be the statistically most plausible causal SNP of the *BANK1* association to SLE in the EUR sample, as previously suggested. It has been remarked that, as a result of the LD status among the markers in ‘block two–group five’ (intraBK = 0.96) (Supplemental Figure S2B), we would expect a similar effect with all of them. We observed that from the conditioning on each of the three SNPs, rs10028805, rs13136297, and rs4411998, the same results were obtained, (=0.99). However, traces of association with a low-frequency variation remained (Table 1, Supplemental Table S1A, and Figure 2b). Only after the conditioning on rs10028805 and three low frequency variants, rs148572654, rs62321740, and 4:102945562:GT:G, did the signals of association in the *BANK1* completely disappear (Table 1, Supplemental Table S1A, and Figure 2c).

In AA, the markers that were significantly associated (10^−8^) were located in the same regions as in EUR, intron one, and intron two–exon two–intron three (Table 2 and Supplemental Table S1B). Intron one was split in two haplotypic blocks, one and two. In the second block, markers were distributed in four linkage disequilibrium groups (one, two, three, and four) and the best hits were in the third group (equivalent to EUR ‘block one–group three′), SNP rs4699262 (Pcorr = 3.19E–08). Region intron two–exon two–intron three was divided into three haplotypic blocks (three, four, and five) containing 12 linkage disequilibrium groups (Supplemental Table S1B). The best hits were in the fourth haplotypic block, group 13 (partially equivalent to EUR group six), with the best-hit rs17200824 (Pcorr = 9.24E–09) and the branch-point site SNP rs17266594 (intron one) and rs10516487 (R61H, exon two).

Note that AA group eight, partially equivalent to EUR group six, containing the best-hits, did not contain associated markers at the genome-wide association study (GWAS) level in AA (SNP rs10028805 had a *p* value = 4.72E–04) (Supplemental Table S1B), and was independent of group 13 (AAgroups_8_13 = 0.18 and EURgroups_5_6 = 0.70).

No marker, including the best hit in the AA population, rs17200824, was able to completely eliminate the associations of all of the others, (Table 3 and Supplemental Table S1B). Only by conditioning on rs17200824 and two variants, rs4295265 and rs149302668, did all of the signals of the association in *BANK1* disappear (Table 3, Supplemental Table S1B, and Figure 5c). Note that rs4295265 (Pcorr = 3.89E–07) was located on intron seven at 2319 bp upstream to the exonic SNP rs3733197 (Pcorr = 1.44E–07) (exon seven, A353T ankyrin domain), described as SLE associated one. Thus, the elimination of the genetic association in AA required conditioning on the markers located in at least two separate blocks, suggesting two independent but closely related signals, as well as suggestive signals in the intron [7].

**Figure 6:**
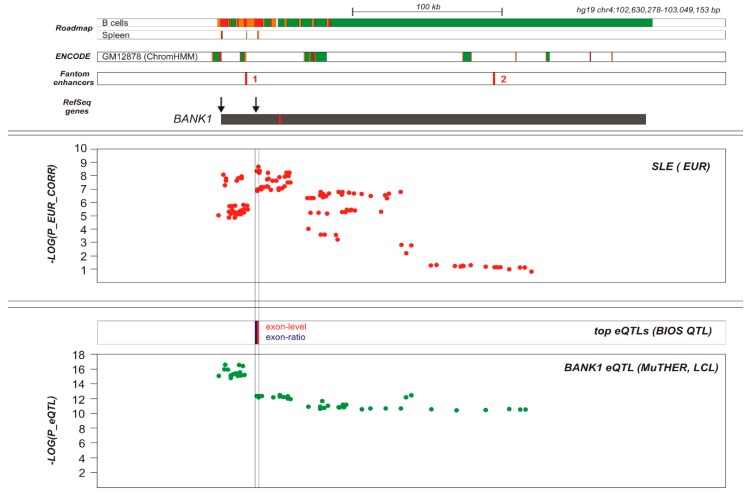
*BANK1* gene regulation and systemic lupus erythematosus (SLE) association map. Functional map of *BANK1* locus. RefSeq transcripts are shown. The alternative exon is red-highlighted. Functional elements are shown according to ChromHMM segmentation for Roadmap Epigenomics project (B cells, spleen) and ENCODE (GM12878 lymphoblastoid cell line [LCL] cell line). Transcribed areas, promoter, and enhancer marks are depicted as green, red, and orange rectangles, respectively. Fantom5 tissue-specific transcribed enhancers loci are shown as enhancer 1 (hg19 chr4:102728165-102728530) specific to spleen, blood, and B-lymphocytes; enhancer 2 (hg19 chr4:102894116-102894225) specific to lymph node, esophagus, B-lymphocytes, melanocytes, and basophils. Shown below is the SLE association plot for European data and expression quantitative trait locus (eQTL) map for *BANK1* gene in blood (top markers shown for exon-ratio and exon-level eQTL, according to BIOS [whole blood data] and to MuTHER [LCL data]).

We then performed a trans-racial ‘EUR–AA’ meta-analysis. We selected 165 markers that showed association *p* values less than 0.05 in both of the EUR and AA samples (Supplemental Table S2). The set of meta-analysis best signals for association were located on the region comprising intron two, exon two, and intron three. In EUR, it coincided with haplotypic block two containing linkage disequilibrium groups five and six, while in AA, the same region was divided into three haplotypic blocks containing seven linkage disequilibrium groups (5, 6, 7, 8, 9 10, and 13). Note that the markers in the EUR linkage disequilibrium groups five and six, in the AA sample, were divided into groups of 5, 8, 9, and 10, and 6, 7, and 13 (Table 5, Supplemental Table S2, and Supplemental Figure S3B). The meta-analysis of the SNP rs10028805 in groups EUR 5 and AA 8 showed OR = 0.8167, CI = 95% (0.7712, 0.8648), and P_val_ = 4.33E–12. Interestingly, rs10028805 had a P_val_ = 4.71 E–04 in the AA sample, clearly under the accepted GWAS threshold. It should be noted that the linkage disequilibrium group AA 13 (and EUR 6) contained the rs71597109, best hit of the meta-analysis, OR = 0.7898, CI = 95% (0.7423, 0.8404), and P_val_ = 6.86E–14. In addition, SNP rs71597109 shared, with group AA 13, rs17200824 and the markers described as the best causal hits of the *BANK1* association to with SLE, such as the branch-point site SNP rs17266594 on intron two, and the R61H rs10516487 on exon two. We then performed an eQTL analysis using various sources of data available, [8,9,10,11], as described. The analysis showed that several associated and non-associated SNPs had eQTL effects. In fact, all of the strongly associated SNPs were eQTLs covering a region from the promoter of *BANK1* to UTR-3′/downstream region. However, the strongest eQTLs, according to the BIOS browser10, were localized to intron two, close to exon two, exon two, and intron three, correlating with the most associated SLE signals at the GWAS significance level. These eQTLs were rs13136297 (Cis-eQTL Exon-ratio *p* = 5.29E–39) and rs10028805 (Cis-eQTL exon-level *p* = 2.74E–52), on the European haploblock two, linkage group five, and the AA haploblock three, linkage group eight. Importantly, risk was associated with an increased expression of *BANK1* (Supplemental Table S3 and Figure 6), and importantly, the effects were directly observed in the RNA-Seq data at the exon level of the expression. Using the BIOS data, we also identified a cis-meQTL, specifically rs6833764 containing CpG cg01116491 (*p* = 3.64E–35), located in the European haploblock one linkage group one; this SNP is however not associated with SLE in AA (Supplemental Table S3).

In order to better define the functional effects on the *BANK1* gene expression, we performed an analysis of the ENCODE tracks and investigated the possible causes for the eQTLs. This analysis revealed that the number of annotations in the *BANK1* SNPs increased from the promoter, peaking in the region of association of SLE, centered in intron two and exon two. Using the meta-analysis data, these annotations can be observed in the Supplemental Table S4 (see the various sheets for transcription factor binding site, histones, and junctions). These annotations showed an enrichment of the splice junctions (*p* value = 1.98E–08, Fisher’s exact test), and in various histone marks, particularly histone three lysine 36 27 trimethylation (H3K27me3, *p*-value = 1.20E–10) (Supplemental Table S4), in the lymphoblastoid cell line Gm12878, further supporting a clear regulatory *BANK1* region in this area. The activating histone marks, such as H3K4me1, H3K4me3 in peripheral, and cord blood B cells and CD56+ NK cells, covered the whole associated region. Some spots were also observed in the monocytes and CD34+ hematopoietic stem cells (HSC), but these were clearly less abundant across the region. In addition, the best-hits were enriched for the sites of binding of the transcription factor EZH2 in the Gm12878 cell line (*p* value = 2.99E–44). One transcription factor binding site for RUNX3, an activator of the transcription of B cells, was observed in the GM12878 lymphoblastoid cell line, where SNP rs13106926 is located (Supplemental Table S4) (UCSC genome browser, coordinates chr4: 102,739,791–102,739,791 of GRCh37/Hg19) (Supplemental Material) 14. This SNP is also found within an active histone mark, histone 3 lysine 27 acetylation mark (H3K27Ac), according to the UCSC browser14. This SNP is an eQTL in lymphoblastoid cell lines, according to the Harvard browser15, but no records were obtained on the BIOS browser. This SNP is located within the European haplotypic block two, group six, and the AA block three, group six, and is strongly associated with SLE (Supplemental Table S2).

Combining all of the information, our data supports the functional effects limited to the regions of intron two, exon two, and intron three, in both European and African Americans. As expected, the AA population shows a more dispersed haplotypic structure than Europeans, separating the haplotypic block two containing linkage disequilibrium groups five and six, into three haplotypic blocks (three, four, and five) with seven linkage disequilibrium groups (5, 6, 7, 8, 9 10, and 13) (Supplemental Table S2 and Supplemental Figure S3B).

## 3. Discussion

We identified a set of SNPs in intron one and surrounding exon two, clearly associated with SLE in the two studied populations, namely European and African Americans. In our current study, we did not present in vitro functional tests for these SNPs. We carried out a bioinformatics analysis and showed that all of the strongest associated SNPs are *BANK1*-associated eQTLs, and associate with the enrichment of various epigenetic marks known to associate with pre-mRNA co-transcriptional exon splicing [8,12]. Furthermore, the data supports, in particular regions containing three SNPs with potential functional properties, rs13136297 (Cis-eQTL exon ratio), rs10028805 (Cis-eQTL exon level), and rs13106926 (eQTL), the latter located within the RUNX3 TFBS and an active regulatory element histone mark in a lymphoblastoid cell line. The combined presence of the histone marks observed, and the enrichment of the splice junctions further support such co-transcriptional splicing as a mechanism regulating the splicing of exon two of *BANK1* involving a higher level of regulation of the chromatin topography of the locus. This is also supported by the eQTL data obtained from BIOS browser. These authors analyzed the functionality of exon ratio cis-eQTLs in mRNA splicing, showing an enrichment of splicing events and a close proximity to the splicing acceptor and donor sites [9].

Recently, it was shown that the SNPs of *BANK1*, associated with SLE and identified through targeted sequencing, increased the expression of the *SLC39A8* gene in the LPS stimulated CD14+ monocytes. *SLC39A8* is a gene located immediately downstream of *BANK1*. The authors utilized the GEUVADIS database, based on the expression data in the lymphoblastoid cell lines (LCLs) and HapMap SNPs [10]. We have used the Zhernakova data [9], based on whole blood RNA sequencing information from a large number of European individuals, and still, not all of the SNPs have been found and the complete information is not fully available. The SNP shown by Raj et al. [11] to be strongly associated with SLE and an eQTL for *SLC39A8* is actually a weak eQTL for this gene, and the effect is probably related to its LD with SNPs in the promoter of *SLC39A8*. The main eQTL peak for *SLC39A8* is located within the promoter of that gene [9], not *BANK1*. We have analyzed our data for a putative association of the *SLC39A8* locus and SLE, and did not observe any evidence of this association (data not shown). It disproves the involvement of *SLC39A8* in the SLE pathogenesis, proposed by Raj and co-authors [11].

Our genetic analysis and the fact that risk was associated with an increased *BANK1* expression at the exon level, supports our original postulate that the increased expression of the exon two coded domain of *BANK1* may be involved in the development of autoimmunity, and that this expression and the splicing of exon two are epigenetically regulated.

We show the enrichment of given epigenetic marks, such as H3K27me3 and EZH2, in the *BANK1* associated SNP region. Furthermore, the presence of such epigenetic marks supports the observation of the eQTLs in the SLE-associated SNPs, and that the region could function as an alternative promoter. In fact, this might be the case, as the *BANK1* gene has two transcription initiation sites, and the first description of the gene had missed the first initiation site [2].

We found one SNP lying with five nucleotides within the binding site of RUNX3, a transcriptional activator. However, this TFBS is only observed in the lymphoblastoid cell lines and not primary B cells. Importantly, the RUNX3 transcriptional activity is induced during the Epstein–Barr virus (EBV) infection of B cell immortality, leading to the repression of RUNX1, with both factors considered as interesting transcriptional regulators of autoimmunity [13,14,15]. An infection with EBV has been considered as an environmental trigger of SLE [16,17]. Nevertheless, we do not know if RUNX3 is induced in lupus B cells or if the modifications in the RUNX3 transcriptional activity have a role in the *BANK1* expression. Thus, the role of the SNP rs13106926 as an eQTL, associated with SLE and the RUNX3 transcriptional activity in EBV infected cells is plausible, but needs to be experimentally tested. Another transcription factor with numerous sites across the region, particularly in intron one, was the histone methyltransferase EZH2, a member of the polycomb repressive complexes that promote silencing by catalyzing posttranslational modifications of histones. EZH2 catalyzes the histone three, lysine 27 trimethylation rich in transcriptional start sites of the repressed genes. This transcription factor has been shown to protect germinal center B cells and impede the mutation of the activation-induced cytidine deaminase, an enzyme key in the class switch recombination of the immunoglobulin heavy chain locus [18]. Its role in SLE B cells and the induction of *BANK1* is also not known, but a role for *BANK1* in the class switch recombination was recently suggested in the mouse [6].

Alternative splicing involves various chromatin modifications, including histone modifications [19]. These changes may function as scaffolds or adaptors for the binding proteins that are part of the splicing machinery. In particular, an important protein is RNA Pol II, in charge of the elongation, which interacts with histone modifiers to recruit SR proteins [19]. These proteins are particularly efficient during co-transcriptional splicing events. In addition, the modulation of H3K36me3 or H3K4me3 levels by the overexpression or downregulation of their respective histone methyltransferases, changes the tissue-specific alternative splicing pattern in a predictable fashion [20]. Studies have shown that a chromatin adaptor protein (CHD1) has a chromodomain that specifically recognizes H3K4me3 and interacts with specific small RNA proteins28, particularly components of the U2 snRNP complexes.

We reported [7] that the protective allele (61H) of the associated coding SNP rs10516487 led to the formation of a site for the splicing trans-factor Srp40, suppressing splicing of exon two and, hence, of the FL isoform. Thus, the differences in the expression of the D2 and FL isoforms of *BANK1* lie behind the risk for SLE, and it is feasible that the co-transcriptional regulation of exon two splicing, combined with the presence of R61, promotes the expression of the FL isoform and enhanced signaling in susceptible individuals. Our data and our animal work fully support an increase in the FL *BANK1* expression, with increased *BANK1*-dependent toll-like receptor 7/9 signaling. Interestingly, the RNA splicing events are controlled by the abundance of SR proteins modulated by the signaling pathway activity, including the pathways controlled by the phosphatidyl-inositol-3 (PI3K) kinase, a key molecule in B cell signaling [21], which regulates the phosphorylation of the serine of the Srp40 trans-activator protein. Therefore, the presence of co-transcriptional alternative splicing modifiers supports our previous data.

## 4. Materials and Methods

### 4.1. Sample Collection and Genotyping

We used GWAS data from 5478 individuals of European ancestry, including 4254 SLE patients and 1224 controls genotyped using the Illumina HumanOmni1_Quad_v1-0_B chip [12]. The U.K. subjects with SLE in the study were recruited, with the study having obtained ethical approval from the London Ethics Committee. Individuals were invited into the study and given information sheets as well as verbal explanations of what the research entailed. For those individuals willing to participate, informed written consent was obtained. The recruitment in continental Europe and Canada were subject to local reviews and ethical approval. Copies of the relevant supporting documentation were sent to the investigators at King’s College at the commencement of the study [12]. In order to increase the number of controls, data from the European controls that were genotyped on the same platform were obtained from the dbGaP database (http://www.ncbi.nlm.nih.gov/gap), including the DCEG Imputation Reference Dataset (phs000396.v1.p1; 1175 individuals), the GENIE UK-ROI Diabetic Nephropathy GWAS (phs000389.v1.p1; 903 individuals), and the High Density SNP Association Analysis of Melanoma (phs000187.v1.p1; 1047 individuals). In total, the initial dataset consisted of 4254 SLE patients and 4349 controls. We used a set of individuals with African American (AA) ancestry, collected from several of the SLEGEN groups and from the Oklahoma Medical Research Foundation, following ethics committee approval and informed consent [22]. The African American samples were genotyped with the same Illumina platform HumanOmni1_Quad_v1-0_B chip as the European individuals (unpublished data).

### 4.2. Data Filtering

In order to obtain a quality-controlled working dataset satisfying current state-of-the-art criteria for the association studies, data filtering was conducted using PLINK v1.07 [23], applying the following criteria: minimum total call rate per sample of 90% (18 individuals were eliminated), minimum call rate per marker of 98%, minor allele frequency (MAF) threshold of 0.01%, Hardy–Weinberg Equilibrium (HWE) *p*-value at a minimum of 0.0001 for cases, and 0.01 for controls, as well as a final cutoff *p* value of 0.00001 for differential missingness in the no-call genotypes between the cases and controls.

### 4.3. Stratification Analysis

To assess the genetic diversity in the EUR data set, markers (linkage equilibrium *r*^2^ < 0.1) with maximum differences in the allele frequencies between the HapMap subpopulations (CEU, MXC, YRI, CHB, EUR, AMR, AFR, and ASN) were selected. A first principal component analysis (without sigma threshold) showed that all of the samples were indeed of European descent. In the next step, the genetic diversity within the European context was examined using 13,222 markers (*r*^2^ < 0.1), with maximum allele differences between the HapMap European subpopulations CEU and TSI (north central and southern Europe, respectively). The principal component analysis was performed with smartpca, EIGENSOFT 4.0 beta package [24]. A set of 60 samples was detected as outliers, using a sigma_threshold = 6.0. This low number of outliers supports the European homogeneity of the sample. An association analysis by logistic regression corrected for stratification with the first 10 principal components resulted in a λGC = 1.05. The cryptic relatedness was assessed using REAP [25], by removing one individual from each sample pair with a kinship-coefficient greater than 0.055. Using this procedure, 248 samples were removed. The final data set contained 573,370 autosomal markers from 8277 individuals (4212 cases and 4065 controls).

The final African American data set contained 2899 individuals, 1761 SLE patients, and 1138 controls. This data set was supplied after QC-filtering, and removing the outliers and ‘closely-related’ samples. For each African American sample, coordinates for the four first PCs were provided.

### 4.4. Statistical Analyses

The allelic association for each marker was tested using the Plink additive logistic regression, testing for the minor allele, as in the literature [23]. The Tracy–Widom test was used to evaluate the significance of the principal components. Association tests included the axes of the 10 most significant principal components as covariates. The dependency of association for the genetic variants with the lowest *p* values were systematically examined by conditional testing. Plots were generated using a modified version of LocusZoom [26]. The haplotypic relations, defined by D’ (four gametes rule), were computed using Haploview version 4.2 [27]. The haploview tagger tool was used (threshold *r*^2^ ≥ 0.8) to select the minimum set of tagging markers containing the allelic variability of the whole *BANK1* region, defining the linkage disequilibrium groups of the markers. The haplotype analyses were carried out with Plink, and the phase was confirmed with BEAGLE version 3.3 [28].

The markers within the region of the *BANK1* gene were selected for imputation with IMPUTE2 [29] using GWAS and 1000Genomes data as reference (http://www.1000genomes.org). Prior to that, the GWAS data set was pre-phased with Shapeit [30], using only the 1000G EUR populations as reference. For imputation, a restrictive QC-filter was applied (SNP genotyping rate ≥99%, sample genotyping rate ≥98%) without restriction of the allele frequencies, in order to include rare and low frequency variants. To ensure a high degree of reliable imputation, a conservative ‘info_value’ threshold of >0.8 for each marker was applied. The same imputation procedure was used with the AA data set, with the only difference being that the AFR 1000Genomes’ populations were used as reference in the Shapeit pre-imputation.

A trans-racial ‘EUR–AA’ meta-analysis was implemented using Metal [31] and Plink. The beta parameter in the logistic regression (natural logarithm of the odds ratio) was used as the effect and the sample size in each study was used as the weighting factor. Two criteria for selecting the markers resulting in the meta-analysis were used, namely: SLE association *p* value < 0.05 values and the same direction of the effect in both studies, and heterogeneity-test *p* value > 0.01.

### 4.5. eQTL/meQTL Analysis

In order to explore the relationship between SLE and the *BANK1* gene expression, we analyzed the expression quantitative trait loci (eQTLs) from several recent databases for lymphoblastoid cell lines (LCLs), cells, or tissues obtained from donors of European ancestry [8,9,10]. MuTHER [32] includes the gene expression data from fat, skin, and LCLs. The BIOS browser [9] (genenetwork.nl/biosqtlbrowser) containing RNASeq data from the peripheral blood derived from 2116 Dutch individuals was used to define the remaining eQTLs of *BANK1*. This data set provides quantitative gene and exon expression data various cis-eQTLs and meQTLs, combined with the cell type and context-specific data. The RTeQTL [33] browser (http://eqtl.rc.fas.harvard.edu) was used to search for information on rs13106926.

### 4.6. Annotation Analysis

We used the genome annotation data assembled under the GenomeRunner project [34] to annotate the set of *BANK1* associated SNPs. Genome annotations include the cell type specific histone modification marks, transcription factor binding sites, splice junction regions, and other regulation-related tracks from the ENCODE and Roadmap Epigenomics consortia [35,36]. The analysis was restricted to the cell type-specific regulatory marks, Gm12878, B-cell lymphoblastoid cell line for the ENCODE data, and the ‘HSC_and_Bcell’ tissue IDs for the Roadmap Epigenomics data. Only the annotations that overlap with at least one SNP are reported.

The enrichment *p* values were calculated using Fisher’s exact test, and were adjusted for multiple testing using the false discovery rate (FDR) method. Briefly, the enriched co-localization of the set of *BANK1* associated SNPs was assessed against the null hypothesis of the random co-localization. To evaluate the random co-localization, a set of all of the SNPs from the *BANK1* region (chr4: 102614068-103075430, the human GRCh37/hg19 genome assembly) was obtained from dbSNP build 138, totaling 8772 ‘background’ SNPs.

## 5. Conclusions

Our analysis of two populations showed that the eQTL SNPs could extend to various portions of the gene, but were indeed centered on the same major region of the *BANK1* gene, surrounding exon two. The genetic association of *BANK1* in AA individuals is particularly strong and supports an important role of the B cell-related pathways in the disease in those individuals. The linkage disequilibrium groups AA 13 and EUR 6 contained, what could be considered the best meta-analysis hits, the SNP rs71597109 and markers described as the best causal hits of the *BANK1* association to SLE, such as the branch-point site SNP rs17266594 on intron two, and the R61H rs10516487 on exon two.

The presence of at least two independent genetic effects in AA shows that *BANK1* contributes to the inheritance of risk through two separate haplotype blocks containing eQTLs.

## Figures and Tables

**Table 1:** Eighteen ankyrin repeats 1 (*BANK1*) tagging markers with an association *p* value lower than 0.05, representing their respective linkage disequilibrium groups in the European (EUR) sample. (*p* *—*p* value conditioning on rs10028805; *p* **—*p* value conditioning on rs10028805, rs148572654, rs62321740, and 4:102945562:GT:G; A*—represents the allele used in the association analysis; HP.BK—haplotypic block; LK.GR—linkage disequilibrium group).

HP.BK	LK.GR	CHR	MK.ID	BP	A1	A2	A*	F.ALL	F.CAS	F.CTR	NMISS	OR	L95	U95	*p*	*p* *	*p* **
1	1	4	rs4518254	102714254	G	T	1	0.42	0.3913	0.4468	8059	0.8256	0.7738	0.8807	6.36E−09	5.52E−02	4.81E−01
1	2	4	rs72602430	102715998	T	TG	1	0.1040	0.1000	0.1081	8111	0.8968	0.8079	0.9956	4.10E−02	3.66E−01	8.95E−01
1	3	4	rs36128945	102719913	A	C	2	0.3051	0.2831	0.3279	8229	0.8541	0.7970	0.9153	7.88E−06	6.06E−01	9.88E−01
1	4	4	rs28496978	102735437	T	A	2	0.0650	0.0641	0.0659	7891	0.8760	0.7679	0.9993	4.88E−02	8.82E−01	5.99E−01
2	5	4	rs10028805	102737250	G	A	2	0.3631	0.3384	0.3887	8277	0.8177	0.7656	0.8735	2.22E−09	NA	NA
2	6	4	rs2080820	102758403	C	A	2	0.2907	0.2668	0.3153	8240	0.8268	0.7709	0.8867	9.87E−08	6.00E−01	8.16E−01
#	7	4	rs17031708	102764358	G	A	2	0.1307	0.1209	0.1409	8259	0.8724	0.7933	0.9594	4.89E−03	4.88E−02	1.45E−01
3	8	4	rs148572654	102764832	G	T	2	0.0195	0.0161	0.0231	8114	0.7551	0.5968	0.9555	1.93E−02	4.08E−03	NA
3	9	4	rs6857428	102792611	T	G	2	0.4496	0.4265	0.4737	8239	0.8442	0.7925	0.8992	1.43E−07	1.84E−01	4.71E−01
3	10	4	rs11939568	102810162	A	G	2	0.3278	0.3094	0.3469	8235	0.8805	0.8233	0.9417	2.04E−04	8.33E−01	7.82E−01
3	11	4	rs10212686	102822654	A	G	1	0.0473	0.0439	0.0508	8183	0.8474	0.7286	0.9856	3.17E−02	3.50E−02	7.42E−01
4	12	4	rs62321740	102824467	A	G	2	0.0339	0.0301	0.0378	8050	0.8172	0.6831	0.9777	2.73E−02	2.62E−03	NA
4	13	4	rs4295265	102835645	C	T	2	0.3179	0.3000	0.3363	8052	0.9009	0.8414	0.9645	2.73E−03	9.34E−01	9.83E−01
4	14	4	rs4099101	102847428	A	G	1	0.1251	0.1203	0.1301	8244	0.8790	0.7985	0.9677	8.57E−03	3.03E−01	8.05E−01
4	15	4	rs33973573	102855806	G	A	2	0.4645	0.4812	0.4472	8210	1.1180	1.0490	1.1910	5.75E−04	9.17E−01	3.50E−01
4	16	4	4:102857352:AT:ATT	102857352	AT	ATT	2	0.0312	0.0274	0.0351	8080	0.7753	0.6447	0.9324	6.85E−03	1.96E−03	1.87E−01
4	17	4	rs2851336	102857563	C	T	2	0.4128	0.4000	0.4261	8120	0.9355	0.8768	0.9982	4.40E−02	5.58E−01	4.58E−01
4	18	4	4:102945562:GT:G	102945562	GT	G	2	0.0109	0.0082	0.0137	8181	0.5969	0.4351	0.8187	1.37E−03	9.43E−03	NA

**Table 2:** Haplotype rs10028805|rs148572654|rs62321740|4:102945562:GT:G association analysis in the EUR sample.

HAPLOTYPE	F.CAS	F.CTR	F.ALL	*p*
1111	0.6212	0.5605	0.5830	3.89E−13
2111	0.3335	0.3810	0.3520	7.64E−08
1121	0.0297	0.0372	0.0340	3.31E−02
1211	0.0155	0.0213	0.0180	5.07E−02

**Table 3:** Fifty *BANK1* tagging markers with association *p* value lower than 0.05, representing their respective linkage disequilibrium groups in the AA (African American) sample. (*p* *—*p* value conditioning on rs17200824; *p* **—*p* value conditioning on rs17200824, rs4295265, and rs149302668; A*—represents the allele used in the association analysis; HP.BK—haplotypic block; LK.GR—linkage disequilibrium group).

HP.BK	LK.GR	CHR	MK.ID	BP	A1	A2	A*	F.ALL	F.CAS	F.CTR	NMISS	OR	L95	U95	*p*	*p* *	*p* **
#	1	4	rs10031210	102714886	A	G	1	0.2233	0.2010	0.2479	2529	0.7563	0.6609	0.8655	4.92E−05	1.37E−01	7.25E−01
1	2	4	rs4699262	102726005	G	A	2	0.1316	0.1090	0.1613	2520	0.6232	0.5271	0.7369	3.19E−08	1.36E−02	2.11E−01
1	3	4	rs35828857	102729253	A	AT	2	0.1633	0.1393	0.1950	2465	0.6727	0.5774	0.7836	3.58E−07	3.52E−02	3.74E−01
#	4	4	rs66638185	102729417	T	C	2	0.1658	0.1388	0.1974	2483	0.6574	0.5654	0.7643	4.92E−08	1.32E−02	3.26E−01
2	5	4	rs13136796	102736743	A	T	2	0.3177	0.2929	0.3459	2494	0.7764	0.6883	0.8757	3.78E−05	4.88E−01	3.36E−01
2	6	4	rs4615176	102737936	C	G	1	0.2351	0.2466	0.2205	2448	1.1790	1.0290	1.3510	1.81E−02	5.38E−01	3.50E−01
2	7	4	rs1421627	102739536	G	A	2	0.4148	0.3953	0.4393	2529	0.8230	0.7345	0.9221	7.84E−04	6.58E−01	6.26E−01
2	8	4	rs13136219	102743687	C	T	1	0.3893	0.4098	0.3623	2466	1.2320	1.0960	1.3850	4.70E−04	2.67E−01	2.10E−01
#	9	4	rs13112246	102743811	T	C	2	0.4072	0.3826	0.4361	2475	0.8045	0.7179	0.9015	1.81E−04	8.76E−01	9.28E−01
#	10	4	rs56857058	102749300	A	AT	1	0.3069	0.3208	0.2837	2515	1.2080	1.0690	1.3650	2.42E−03	3.29E−01	1.21E−01
3	11	4	rs144220121	102750582	G	GT	2	0.1755	0.1895	0.1610	2478	1.2010	1.0320	1.3970	1.78E−02	3.04E−01	4.71E−01
3	12	4	rs11938136	102750726	G	A	2	0.1027	0.1124	0.0925	2501	1.2240	1.0160	1.4750	3.31E−02	2.74E−01	3.51E−01
3	13	4	rs17200824	102752589	A	G	2	0.2281	0.1984	0.2664	2516	0.6787	0.5947	0.7747	9.24E−09	NA	NA
3	14	4	rs7438361	102756142	G	A	2	0.2143	0.2248	0.2000	2427	1.1570	1.0070	1.3290	3.96E−02	6.79E−01	7.94E−01
#	15	4	rs72923462	102758014	G	A	2	0.0677	0.0725	0.0571	2427	1.2680	1.0070	1.5980	4.37E−02	2.21E−01	2.50E−01
#	16	4	rs72923467	102760424	G	A	2	0.0810	0.0871	0.0692	2475	1.2780	1.0340	1.5780	2.29E−02	1.65E−01	1.32E−01
4	17	4	rs4607219	102773838	T	C	1	0.2475	0.2619	0.2327	2473	1.1820	1.0370	1.3460	1.21E−02	6.01E−02	1.54E−01
4	18	4	rs34166099	102778580	C	A	2	0.0427	0.0364	0.0510	2428	0.6751	0.5069	0.8993	7.23E−03	1.87E−01	8.52E−01
4	19	4	rs4426778	102780724	G	A	1	0.2772	0.2893	0.2658	2512	1.1400	1.0060	1.2910	3.93E−02	2.07E−01	4.42E−01
4	20	4	rs35201947	102781331	C	T	2	0.2026	0.1792	0.2329	2427	0.7211	0.6268	0.8297	4.87E−06	1.83E−01	7.15E−01
4	21	4	rs34814827	102787117	C	T	2	0.3850	0.3661	0.4046	2513	0.8499	0.7589	0.9518	4.87E−03	5.13E−01	6.53E−01
4	22	4	rs4493533	102820684	C	T	1	0.4461	0.4663	0.4251	2496	1.1790	1.0540	1.3190	3.99E−03	1.67E−01	5.78E−01
4	23	4	rs34612070	102832696	TC	T	2	0.1222	0.1014	0.1469	2429	0.6586	0.5543	0.7826	2.07E−06	5.30E−02	5.63E−01
4	24	4	rs4295265	102835645	C	T	2	0.1107	0.0897	0.1344	2444	0.6206	0.5161	0.7461	3.89E−07	4.97E−03	NA
5	25	4	rs3733197	102839287	G	A	2	0.1827	0.1571	0.2150	2516	0.6762	0.5845	0.7824	1.44E−07	8.90E−03	9.33E−01
5	26	4	rs13119516	102841605	G	A	2	0.0295	0.0238	0.0365	2505	0.6088	0.4325	0.8570	4.44E−03	1.96E−01	7.32E−01
5	27	4	rs36073662	102841949	G	GA	2	0.3743	0.3584	0.3892	2485	0.8659	0.7716	0.9717	1.44E−02	5.73E−01	4.27E−01
#	28	4	rs59723463	102857343	G	GAT	2	0.4205	0.4372	0.3982	2445	1.1740	1.0470	1.3150	5.89E−03	2.33E−01	5.17E−01
#	29	4	rs2851336	102857563	C	T	2	0.4564	0.4414	0.4702	2452	0.8779	0.7830	0.9844	2.57E−02	5.68E−01	5.43E−01
6	30	4	rs150123613	102879041	G	A	2	0.0114	0.0137	0.0081	2424	1.8240	1.0030	3.3170	4.88E−02	8.45E−02	1.10E−01
6	31	4	rs72916176	102881821	T	G	2	0.1235	0.1335	0.1081	2432	1.2560	1.0530	1.4970	1.12E−02	1.06E−01	1.64E−01
6	32	4	rs72929915	102890546	A	G	2	0.1616	0.1731	0.1451	2423	1.2110	1.0340	1.4180	1.75E−02	5.19E−02	1.44E−01
6	33	4	rs976654	102898570	A	C	2	0.2763	0.2478	0.3088	2502	0.7336	0.6462	0.8328	1.69E−06	6.37E−03	2.29E−01
6	34	4	rs1027013	102904490	G	A	2	0.1700	0.1433	0.2018	2526	0.6497	0.5581	0.7564	2.69E−08	6.41E−03	6.95E−01
6	35	4	rs2851318	102915668	A	T	1	0.3250	0.3400	0.3107	2446	1.1450	1.0150	1.2920	2.76E−02	1.45E−01	6.21E−01
6	36	4	rs6817137	102918724	G	A	2	0.1192	0.1299	0.1061	2496	1.2300	1.0300	1.4690	2.26E−02	1.91E−01	2.83E−01
6	37	4	rs2903274	102919138	T	C	2	0.4800	0.4553	0.4940	2500	0.8204	0.7320	0.9195	6.71E−04	1.57E−02	2.34E−01
6	38	4	rs6532981	102927216	T	C	2	0.2169	0.2314	0.2005	2470	1.1850	1.0320	1.3600	1.64E−02	8.45E−02	3.02E−01
6	39	4	rs6532982	102927310	A	G	2	0.1763	0.1906	0.1593	2529	1.2200	1.0520	1.4150	8.47E−03	5.84E−02	1.72E−01
#	40	4	rs1486571	102938040	A	T	1	0.1934	0.1725	0.2168	2415	0.7597	0.6538	0.8827	3.32E−04	4.79E−02	7.27E−01
7	41	4	rs11412036	102942316	G	GA	1	0.0763	0.0626	0.0917	2419	0.6614	0.5320	0.8222	1.97E−04	3.86E−02	3.06E−01
7	42	4	rs2850390	102943996	A	C	1	0.1462	0.1247	0.1693	2529	0.7007	0.5961	0.8237	1.62E−05	1.85E−02	6.68E−01
7	43	4	rs2631268	102948730	G	T	2	0.3030	0.3151	0.2852	2530	1.1470	1.0150	1.2950	2.74E−02	5.63E−02	3.14E−01
7	44	4	rs3133078	102952819	C	A	1	0.1673	0.1467	0.1890	2440	0.7383	0.6317	0.8628	1.36E−04	4.67E−02	7.79E−01
7	45	4	rs149302668	102955310	G	A	2	0.0135	0.0114	0.0182	2507	0.6105	0.3773	0.9876	4.44E−02	2.91E−02	NA
#	46	4	rs11326096	102968589	CT	C	1	0.3067	0.2886	0.3298	2436	0.8334	0.7370	0.9423	3.65E−03	1.92E−01	6.33E−01
8	47	4	rs2850397	102970744	G	T	1	0.2362	0.2135	0.2629	2409	0.7759	0.6785	0.8873	2.10E−04	5.74E−02	4.17E−01
8	48	4	rs2658535	102971016	G	A	1	0.3901	0.3674	0.4214	2454	0.8118	0.7237	0.9107	3.78E−04	6.30E−02	6.12E−01
8	49	4	rs116552800	102974226	A	G	2	0.0386	0.0438	0.0318	2410	1.3840	1.0170	1.8840	3.88E−02	1.23E−01	2.06E−01
#	50	4	rs2129292	102991139	C	G	1	0.3598	0.3400	0.3802	2418	0.8531	0.7572	0.9611	9.00E−03	1.03E−01	8.72E−01

**Table 4:** Haplotype rs4295265|rs149302668 association analysis in the EUR sample.

HAPLOTYPE	F.CAS	F.CTR	F.ALL	*p*
11	0.8999	0.8464	0.8760	3.17E−08
12	0.0112	0.0179	0.0130	5.21E−02
21	0.0889	0.1357	0.1110	4.02E−07

**Table 5:** The EUR–African Americans’ (AA) meta-analysis best signals for association located on the region comprising intron two, exon two, and intron three. In the EUR, the association best-hit coincided with haplotypic block two, containing linkage disequilibrium groups five and six; the AA the region was divided into three haplotypic blocks containing seven linkage disequilibrium groups (5, 6, 7, 8, 9 10, and 13). (A*—represents the allele used in the association analysis; FEM—fixed effect model).

MKID	BP	POS	EUR										AA								META						
			A1	A2	A*	H.B	L.G	N	F	F_CAS_	F_CTR_	P_val_	A*	H.B	L.G	N	F	F_CAS_	F_CTR_	P_val_	OR_EUR_	OR_AA_	OR_FEM_	L95_FEM_	U95_FEM_	P_val_	HetP_val_
rs13136796	102736743	I2	A	T	2	2	6	8227	0.2874	0.2634	0.3123	7.36E−08	2	3	6	2494	0.3177	0.2929	0.3459	3.78E−05	0.8244	0.7764	0.8119	0.7641	0.8627	2.05E−11	0.31
rs66976837	102736943	I2	CTTGTT	C	2	2	6	8228	0.2874	0.2633	0.3124	6.66E−08	2	3	6	2495	0.3176	0.2929	0.3455	4.13E−05	0.8239	0.7774	0.8118	0.7640	0.8626	1.97E−11	0.32
rs10028805	102737250	I2	G	A	2	2	5	8277	0.3631	0.3384	0.3887	2.22E−09	1	3	8	2528	0.3930	0.4139	0.3665	4.72E−04	0.8177	0.8137	0.8167	0.7712	0.8648	4.33E−12	0.87
rs4615176	102737936	I2	C	G	2	2	5	8190	0.3699	0.3452	0.3955	2.33E−09	1	3	5	2448	0.2351	0.2466	0.2205	1.81E−02	0.8177	0.8482	0.8234	0.7759	0.8738	1.83E−10	0.43
rs4411998	102738147	I2	T	C	2	2	5	8213	0.3639	0.3395	0.3892	3.77E−09	1	3	8	2502	0.4029	0.4237	0.3763	6.26E−04	0.8198	0.8177	0.8193	0.7736	0.8677	9.57E−12	0.88
rs2052445	102738722	I2	A	G	2	2	6	8232	0.2877	0.2636	0.3126	7.45E−08	2	3	6	2495	0.3175	0.2926	0.3454	4.19E−05	0.8245	0.7777	0.8123	0.7645	0.8631	2.25E−11	0.32
rs5860695	102738856	I2	TA	T	2	2	6	8203	0.2882	0.2641	0.3132	7.84E−08	2	3	6	2495	0.3175	0.2926	0.3454	4.19E−05	0.8246	0.7777	0.8124	0.7645	0.8633	2.37E−11	0.32
rs35388091	102739452	I2	C	T	2	2	6	8255	0.2890	0.2648	0.3140	5.36E−08	2	3	7	2505	0.4146	0.3946	0.4381	9.61E−04	0.8229	0.8254	0.8236	0.7758	0.8743	2.06E−10	0.79
rs1421627	102739536	I2	G	A	2	2	6	8272	0.2899	0.2663	0.3143	9.40E−08	2	3	7	2529	0.4148	0.3953	0.4393	7.84E−04	0.8138	0.8230	0.8164	0.7686	0.8671	4.24E−11	0.88
rs13106926	102739791	I2	A	G	2	2	6	8070	0.2900	0.2654	0.3169	1.36E−08	2	3	6	2499	0.3180	0.2931	0.3458	4.12E−05	0.8243	0.7775	0.8121	0.7643	0.8629	2.14E−11	0.32
rs13107572	102739928	I2	C	A	2	2	6	8240	0.2879	0.2638	0.3127	7.19E−08	2	3	6	2498	0.3179	0.2929	0.3458	3.94E−05	0.8243	0.7770	0.8120	0.7642	0.8628	2.07E−11	0.32
rs13107612	102739980	I2	C	T	2	2	6	8240	0.2879	0.2638	0.3127	7.19E−08	2	4	6	2492	0.3186	0.2939	0.3461	5.06E−05	0.8226	0.7798	0.8115	0.7637	0.8623	1.80E−11	0.35
rs71597109	102741002	I2	C	T	2	2	6	8231	0.2894	0.2652	0.3144	5.27E−08	2	4	13	2494	0.2266	0.1969	0.2652	1.02E−08	0.8226	0.6786	0.7898	0.7423	0.8404	6.86E−14	0.02
rs13135381	102743353	I2	A	G	2	2	6	8211	0.2881	0.2637	0.3133	6.80E−08	2	4	13	2499	0.2267	0.1978	0.2643	2.27E−08	0.8278	0.6847	0.7941	0.7463	0.8450	2.62E−13	0.02
rs13136219	102743687	I2	C	T	2	2	5	8187	0.3661	0.3423	0.3909	8.21E−09	1	4	8	2466	0.3893	0.4098	0.3623	4.70E−04	0.8237	0.8117	0.8208	0.7750	0.8693	1.63E−11	0.77
rs55768089	102743698	I2	T	A	2	2	6	8212	0.2877	0.2641	0.3122	1.38E−07	2	4	13	2499	0.2267	0.1978	0.2643	2.27E−08	0.8274	0.6847	0.7938	0.7460	0.8447	2.37E−13	0.02
rs13112246	102743811	I2	T	C	2	2	5	8163	0.3662	0.3421	0.3912	9.33E−09	2	4	9	2475	0.4072	0.3826	0.4361	1.81E−04	0.8241	0.8045	0.8191	0.7736	0.8672	8.13E−12	0.61
rs13137133	102744092	I2	C	T	2	2	5	8205	0.3740	0.3512	0.3977	3.58E−08	1	4	10	2491	0.3050	0.3187	0.2817	2.68E−03	0.8312	0.8285	0.8306	0.7838	0.8802	3.49E−10	0.98
rs1125271	102745985	I2	C	T	2	2	6	8213	0.2877	0.2640	0.3122	1.27E−07	2	4	13	2505	0.2219	0.1924	0.2593	1.42E−08	0.8243	0.6794	0.7904	0.7427	0.8412	9.12E−14	0.02
rs4276281	102746780	I2	A	C	2	2	5	8252	0.3734	0.3505	0.3969	4.04E−08	1	4	10	2481	0.3020	0.3140	0.2795	9.75E−03	0.8318	0.8496	0.8357	0.7886	0.8856	1.40E−09	0.71
rs7698632	102747265	I2	G	C	2	2	5	8224	0.3724	0.3489	0.3968	1.54E−08	1	4	10	2511	0.3065	0.3202	0.2837	2.47E−03	0.8271	0.8278	0.8273	0.7807	0.8766	1.40E−10	0.93
rs12163856	102747927	I2	A	G	2	2	5	8159	0.3750	0.3514	0.3994	1.35E−08	1	4	10	2462	0.3538	0.3634	0.3351	1.89E−02	0.8266	0.8681	0.8362	0.7895	0.8856	1.01E−09	0.5
rs56857058	102749300	I2	A	AT	2	2	5	8213	0.3764	0.3530	0.4006	1.77E−08	1	4	10	2515	0.3069	0.3208	0.2837	2.42E−03	0.8282	0.8278	0.8281	0.7816	0.8774	1.58E−10	0.94
rs17266594	102750922	I2	T	C	2	2	6	8124	0.2863	0.2624	0.3109	1.25E−07	2	4	13	2514	0.2249	0.1962	0.2639	1.26E−08	0.8285	0.6795	0.7936	0.7459	0.8443	1.80E−13	0.01
rs10516487	102751076	E2	G	A	2	2	6	8220	0.2864	0.2624	0.3114	7.34E−08	2	4	13	2518	0.2232	0.1936	0.2604	1.41E−08	0.8273	0.6798	0.7929	0.7452	0.8437	1.53E−13	0.02
rs10516486	102751276	E2	T	C	2	2	5	8277	0.3761	0.3528	0.4002	1.30E−08	1	4	10	2530	0.3089	0.3218	0.2866	3.47E−03	0.8269	0.8333	0.8283	0.7818	0.8775	1.66E−10	0.85
rs34029191	102752201	I3	A	G	2	2	6	8237	0.2912	0.2676	0.3155	1.38E−07	2	4	13	2517	0.2275	0.1981	0.2655	1.22E−08	0.8270	0.6804	0.7924	0.7448	0.8431	1.32E−13	0.02
rs17200824	102752589	I3	A	G	2	2	6	8233	0.2887	0.2651	0.3131	1.15E−07	2	4	13	2516	0.2281	0.1984	0.2664	9.24E−09	0.8268	0.6787	0.7919	0.7444	0.8424	9.88E−14	0.01
rs4637409	102753408	I3	A	G	2	2	6	7966	0.2809	0.2565	0.3062	1.04E−07	2	4	13	2527	0.2221	0.1937	0.2604	1.12E−08	0.8264	0.6771	0.7917	0.7441	0.8424	1.05E−13	0.02
rs34749007	102754507	I3	G	A	2	2	6	8239	0.2889	0.2653	0.3133	1.09E−07	2	4	13	2469	0.2313	0.2033	0.2670	1.05E−07	0.8265	0.6977	0.7961	0.7480	0.8473	6.18E−13	0.04
rs5860696	102755799	I3	CA	C	2	2	5	8004	0.3603	0.3351	0.3863	2.52E−09	1	5	8	2422	0.3906	0.4119	0.3643	8.05E−04	0.8165	0.8183	0.8169	0.7709	0.8656	8.07E−12	0.95
rs4643809	102756099	I3	C	T	2	2	5	8038	0.3611	0.3362	0.3869	5.03E−09	1	5	8	2409	0.3844	0.4051	0.3574	7.77E−04	0.8202	0.8163	0.8193	0.7732	0.8682	1.56E−11	0.89

## Supplementary Materials

Supplementary materials can be found at http://www.mdpi.com/1422-0067/19/8/2331/s1.

## Abbreviations

AA	African Americans
eQTL	expression quantitative trait locus
EUR	Europeans
GWAS	genome-wide association study
meQTL	methylation quantitative trait locus
LD	Linkage disequilibrium
OR	Odds ratio
SLE	Systemic lupus erythematosus
SNP	single nucleotide polymorphism
