# Supplementary figures and images for: Trans-Ethnic Mapping of BANK1 Identifies Two Independent SLE-Risk Linkage Groups Enriched for Co-Transcriptional Splicing Marks
Source: Int J Mol Sci. 2018 Aug 8;19(8):2331. doi: 10.3390/ijms19082331 (PMC6121630; doi:10.3390/ijms19082331)

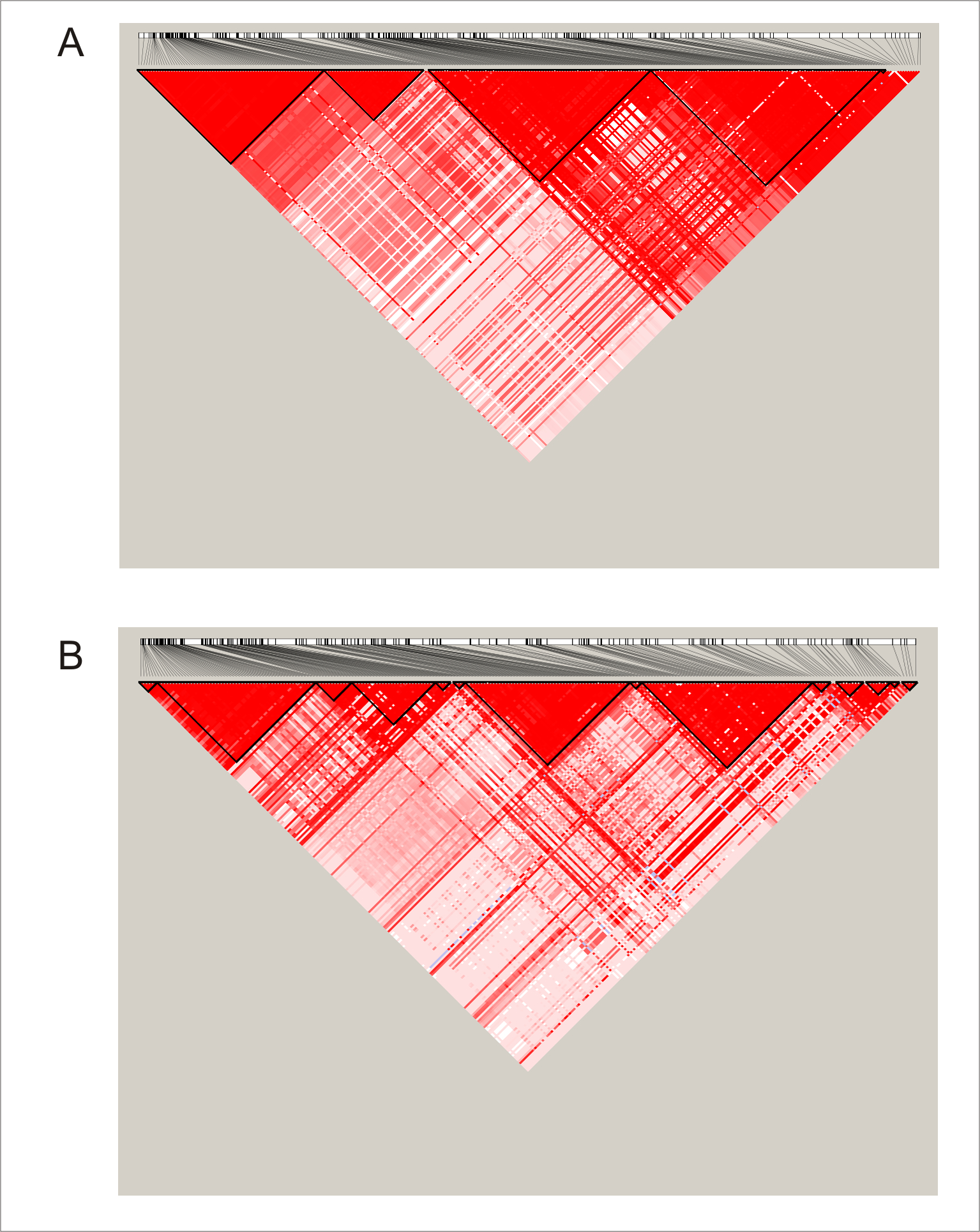

Supplement: Supplementary file 1 [file ijms-19-02331-s001.zip › SupplFigureS1.png]

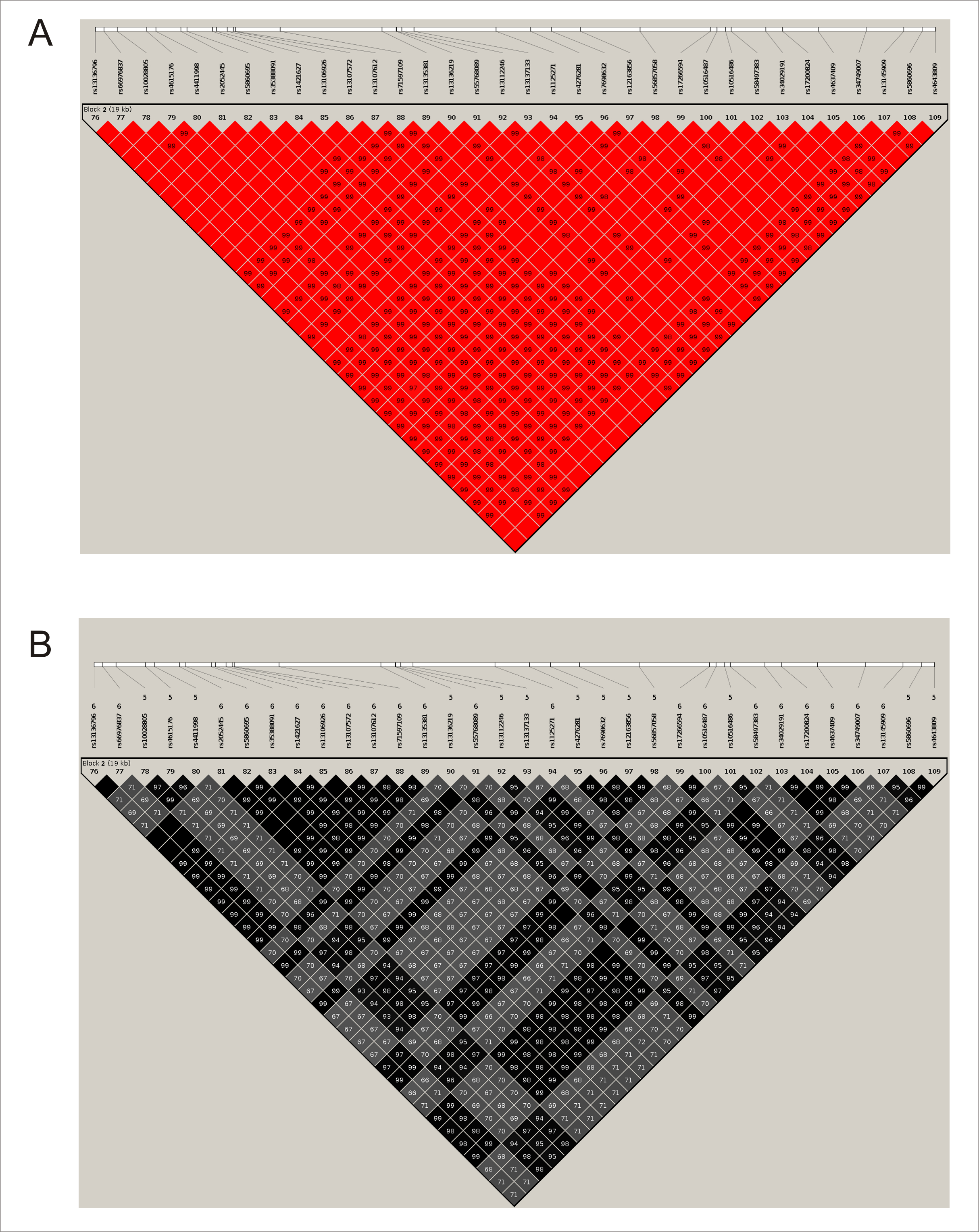

Supplement: Supplementary file 1 [file ijms-19-02331-s001.zip › SupplFigureS2.png]

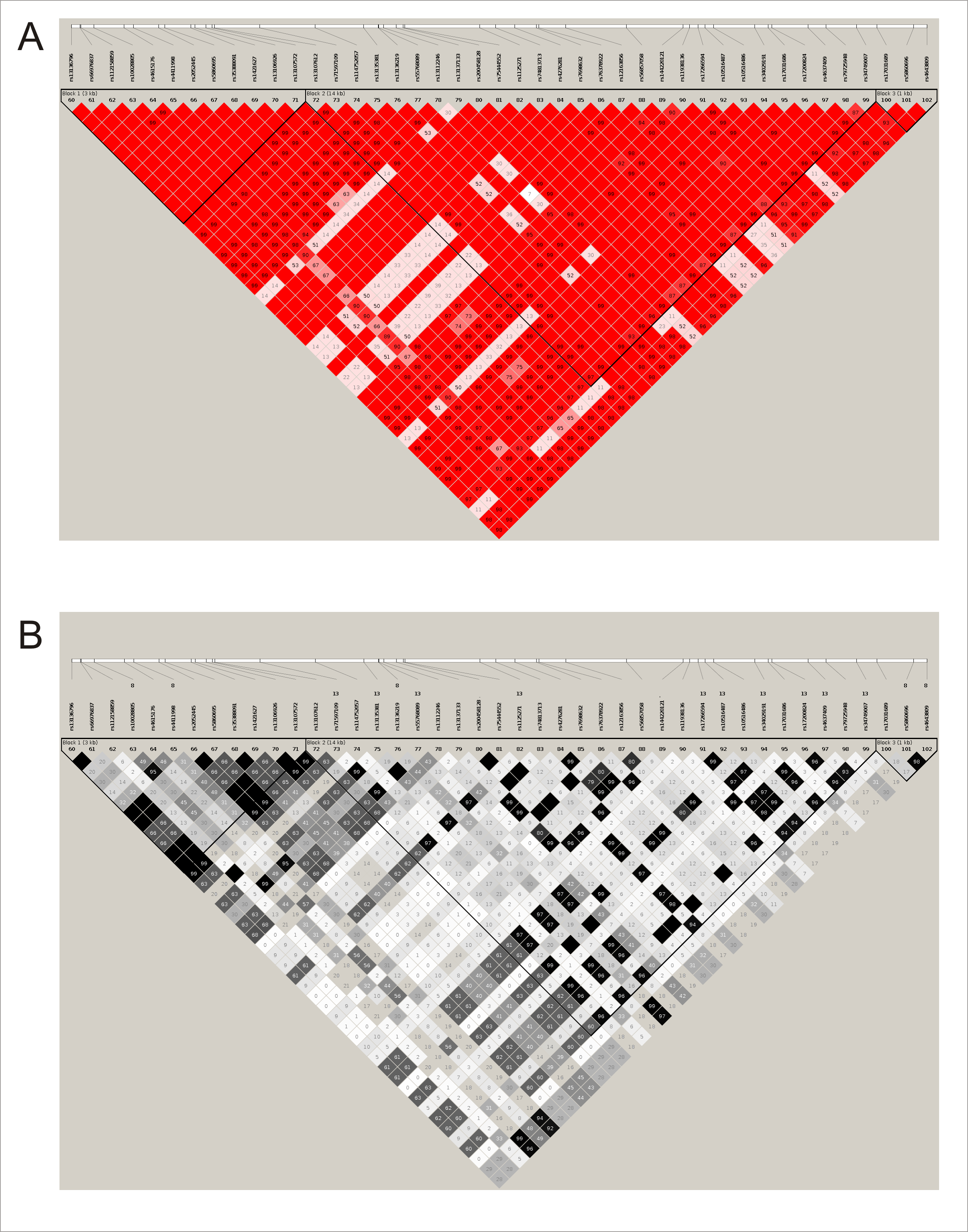

Supplement: Supplementary file 1 [file ijms-19-02331-s001.zip › SupplFigureS3.png]
