# Supplementary material for: Trans-Ethnic Mapping of BANK1 Identifies Two Independent SLE-Risk Linkage Groups Enriched for Co-Transcriptional Splicing Marks
Source: Int J Mol Sci. 2018 Aug 8;19(8):2331. doi: 10.3390/ijms19082331 (PMC6121630; doi:10.3390/ijms19082331)
Supplement: Supplementary file 1 [file ijms-19-02331-s001.zip › SuppFigureLegends.doc]

**Legends to Supplementary Figures.**

**Supplemental Figure S1A:** EUR haplotype blocks. The 307 markers mapping on *BANK1* and with association p values < 0.05 in sample EUR were organized into 4 haplotypic blocks. Calculation of haplotypic blocks was made using D’.

**Supplemental Figure S1B:** AA haplotypic blocks. 260 markers mapping on *BANK1* and with association p values < 0.05 in the sample AA were organized into 14 haplotypic blocks. Calculation of haplotypic blocks was made using D’.

**Supplemental Figure S2A:** EUR-AFA meta-analysis best-signals for asociation were placed on the BANK1 region *intron2, exon2 and intron3*. In EUR sample, it coincided with haplotypic block 2. This Haploview plot showed D’ values.

**Supplemental Figure S2B:** EUR-AFA meta-analysis best-signals for asociation were placed on the BANK1 region *intron2, exon2 and intron3*. In EUR sample, it coincided with haplotypic block 2 containing linkage disequilibrium groups 5 and 6. Number of linakege disequilibrium group in over each marker ID (5 or 6). The group 5, contained rs10028805 (intron2) (Pcorr = 2.22E-09) and rs10516486 (L98L, exon2). For group 6, rs13106926 (intron2) (Pcorr = 1.36E-08) was the best hit; this group also contained the branch-point site SNP rs17266594 (intron2) and rs10516487 (R61H, exon2). Pairwise r² values are shown (between groups 5 and 6 was equals to 0.70).

**Supplemental Figure S3A:** EUR-AFA meta-analysis best signals for association were placed on the region comprising *intron 2, exon 2 and intron 3*. In AFA the region was divided into 3 haplotypic blocks (3, 4 and 5). This Haploview plot showed D’ values.

**Supplemental Figure S3B:** EUR-AFA meta-analysis best signals for association were placed on the region comprising *intron 2, exon 2 and intron 3*. In AFA the region was divided into 3 haplotypic blocks containing seven linkage disequilibrium groups, 5, 6 , 7, 8, 9 10 and 13. The best meta-analysis hit, the SNP rs10028805 was in group EUR 5 and in AFA 8. Linkage disequilibrium group AFA 13 (equivalent to part of EUR 6) contained the rs71597109 sharing group with rs17200824 and markers described as the best causal hits of BANK1 association to SLE the branch-point site SNP rs17266594 (intron2) and rs10516487 (R61H, exon2). Pairwise r² values are shown (between groups 8 and 13 was equals to 0.18).
